# Synaptic Plasticity and Oscillations in Alzheimer’s Disease: A Complex Picture of a Multifaceted Disease

**DOI:** 10.3389/fnmol.2021.696476

**Published:** 2021-06-17

**Authors:** Yuniesky Andrade-Talavera, Antonio Rodríguez-Moreno

**Affiliations:** Laboratory of Cellular Neuroscience and Plasticity, Department of Physiology, Anatomy and Cell Biology, Universidad Pablo de Olavide, Seville, Spain

**Keywords:** Alzheimer’s disease, plasticity, oscillations, spike timing-dependent plasticity, Alzheimer’s disease models, transcranial magnetic stimulation

## Abstract

Brain plasticity is widely accepted as the core neurophysiological basis of memory and is generally defined by activity-dependent changes in synaptic efficacy, such as long-term potentiation (LTP) and long-term depression (LTD). By using diverse induction protocols like high-frequency stimulation (HFS) or spike-timing dependent plasticity (STDP), such crucial cognition-relevant plastic processes are shown to be impaired in Alzheimer’s disease (AD). In AD, the severity of the cognitive impairment also correlates with the level of disruption of neuronal network dynamics. Currently under debate, the named amyloid hypothesis points to amyloid-beta peptide 1–42 (Aβ42) as the trigger of the functional deviations underlying cognitive impairment in AD. However, there are missing functional mechanistic data that comprehensively dissect the early subtle changes that lead to synaptic dysfunction and subsequent neuronal network collapse in AD. The convergence of the study of both, mechanisms underlying brain plasticity, and neuronal network dynamics, may represent the most efficient approach to address the early triggering and aberrant mechanisms underlying the progressive clinical cognitive impairment in AD. Here we comment on the emerging integrative roles of brain plasticity and network oscillations in AD research and on the future perspectives of research in this field.

## Introduction

Brain is constantly changing throughout life with modifications at synaptic levels namely plastic changes that involve morphologic and physiological modifications (Cajal, 1894; Bliss and Collingridge, 1993; Bailey et al., 2015). Brain plasticity is assumed to underlie higher cognitive processes such as memory storage and recall and is disrupted in several brain disorders (reviewed in Kumar, 2011). From the second half of the past century, researchers have revealed that the core neurophysiological basis of memory are most probably activity-dependent changes in synaptic efficacy, such as long-term potentiation (LTP) and long-term depression (LTD) (Bliss and Collingridge, 1993; Kumar, 2011). Such modifications of synaptic activity could promote or disrupt rhythmic electrical activity, which in turn also modulates plasticity evidencing a bi-directional relationship. However, we do not know yet in detail the dynamics of neuronal activity in the brain that give rise to long-lasting experience traces responsible of memory in a healthy condition. In the mammalian neocortex, information processing and plasticity rely on timing precision of neuronal activity within neuronal networks (Buzsáki, 2011). As such, functional neuronal networks emerge from the circuitry established by direct synaptic contact between neurons and indirect feedforward and feedback connections from intercalated neurons, whose recruitment, strength and excitability contribute to the formation and dissolution of neuronal ensembles (Buzsáki, 2010). Brain rhythms emerge from proper activity entrainment of this functionally orchestrated circuitry and are generally (but not exclusively) framed by inhibitory interneurons (Fisahn et al., 1998; McBain and Fisahn, 2001; Fries et al., 2007; Cardin et al., 2009; Goutagny et al., 2009; Verret et al., 2012; Amilhon et al., 2015). Thereby, it has been observed that activity-dependent modulation of perisomatic inhibitory strength effectively influences the participation of single principal cortical neurons (PN) to cognition-relevant network rhythms. For instance, potentiation of feedforward perisomatic inhibition in the layer 5 of the primary somatosensory cortex alters the temporal association of PN during γ-oscillations (Lourenço et al., 2020) and, in healthy humans, visual HFS induces LTP-like neuroplastic changes in visual evoked potentials that enhances theta band power and inter-trial phase coherence (Hamilton et al., 2020). Synchronization between inhibitory neurons is promoted by gap junctions and it is known that stronger coupling of gap junctions leads to plastic changes that regulate oscillations and propagate transient information (Pernelle et al., 2018). Thus, plasticity modulates several features of brain rhythms.

Underlying rhythmic activity within the network also conditions plastic processes. For instance, spike timing-dependent plasticity (STDP), a ubiquitous Hebbian learning rule (Hebb, 1949; Feldman, 2012) in which synaptic modification depends on the precise order of pre- and postsynaptic spiking in a time windows of a few tens of milliseconds (Feldman, 2012), is subjected to control from external inputs. STDP has been found in all of the species in which has been studied (from insects to humans), but it may vary with the specific cell and synapse type as well as with the developmental stage (see Markram et al., 2012). It is possible that firing correlations between local neurons determine whether plasticity will occur, whereas the sign of that plasticity might be determined by information encoded in the timing of an external input relative to the local network dynamics (Kwag and Paulsen, 2009). There is also evidence that basal and apical dendritic synaptic plasticity and spike excitability are facilitated at different theta oscillation phases in a compartmental fashion (Law and Leung, 2018). Additionally, changes in theta-gamma oscillations that appear during HFS to induce LTP may predict whether successful LTP will occur or not (Kalweit et al., 2015). Moreover, somatostatin-positive interneurons (SST) seems to play a pivotal role in hippocampal oscillogenesis mainly supporting the theta rhythm (Park et al., 2020). In particular, hippocampal theta-nested gamma oscillations observed during spatial memory processing have been shown to support the induction of LTP (Buzsáki, 2002; Park et al., 2020). At the same time, some forms of STDP-like have been found altered in Alzheimer’s disease (AD) patients (Di Lorenzo et al., 2018) and cortical LTP disruption has been proposed like a central mechanism of AD that is independent from the age of onset of the disease (Di Lorenzo et al., 2016).

Plasticity processes also participate in how the brain reacts to lesions and injuries (Bach-y-Rita, 2003; Chen et al., 2010; Hill et al., 2011; Sandvig et al., 2018) and its loss could lead to devastating consequences such as in AD. AD is a progressive multifaceted neurodegenerative disorder for which no disease-modifying treatment exists. To date only five drugs have been approved for clinical use to treat the disease with limited effectiveness (see Isla et al., 2021). The aberrant accumulation of amyloid-beta peptide 1–42 (Aβ42), hyperphosphorylated Tau into neurofibrillary tangles and cognitive decline constitute the histological and pathophysiological hallmarks of the disease, respectively (reviewed in Frere and Slutsky, 2018). The amyloid cascade hypothesis posits Aβ aggregates as a major culprit for the toxic effects on brain functions observed in AD, including neuro-inflammation, synaptic and neuronal loss, and tau-associated pathology, and has also been proposed to be responsible of the early cognitive decline observed in AD (Palop et al., 2007; Mucke and Selkoe, 2012; Verret et al., 2012; De Strooper and Karran, 2016; Frere and Slutsky, 2018). However, in light of recent advances, the amyloid cascade hypothesis is currently subjected to critical revisions (see Karran et al., 2011; Karran and De Strooper, 2016; Selkoe and Hardy, 2016; Tolar et al., 2020). While Aβ42 likely plays a major role during early stages of the disease, it has been proposed that Tau pathology plays a prominent role in the symptomatic (late) stages of AD as a key driver of the neurodegeneration (Hyman, 2011; Holtzman et al., 2016). However, synaptic disturbances has been observed in early stages of the tauopathy in animal models (Crimins et al., 2012) with the dendritic spines proposed as the locus of early tau-mediated synaptic dysfunction (Hoover et al., 2010) and tau oligomers proposed as the toxic conformational state of the protein (Lasagna-Reeves et al., 2011). Additionally, in AD, the cognitive impairment goes hand-in-hand with the disruption of neuronal network activity, and the severity of the cognitive decline correlates with the degree of disruption of neuronal dynamics (Stam et al., 2002; Başar, 2013; Guillon et al., 2017; Di Lorenzo et al., 2020).

**FIGURE 1:**
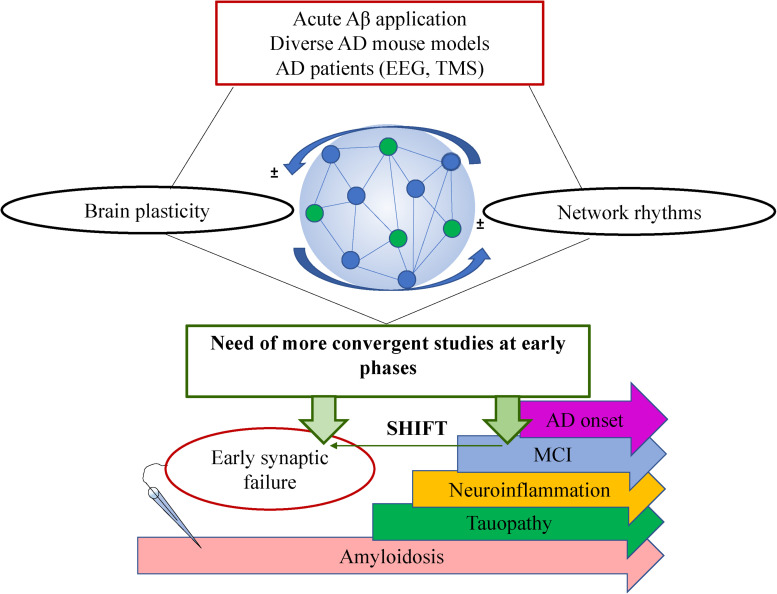
Schematic showing the complexity of AD research with regards to brain plasticity and neuronal network rhythms. A general summary of the type of studies is shown on top (red box), the bidirectional interplay between plasticity and oscillations within the neuronal network is shown in the middle where a very simplified schematic of a neuronal network is shown formed by connected pyramidal cells (blue circles) and interneurons (green circles). The future perspective is shown in the green box claiming a shift of the studies toward earlier time points (green horizontal arrow). On the bottom, there is a schematic timeline showing the most prominent pathological events that takes place during AD progression where electrophysiological recordings at earlier stages have revealed very early synaptic failure on the neuronal circuit’s performance. MCI: Mild cognitive impairment.

From the past 20 years, there is growing evidence indicating that subtle synaptic changes precede neuronal and synaptic loss typical of AD (Selkoe, 2002). Particularly, recent evidences support the notion that functional deviations start much earlier than the onset of solid Aβ42 depositions into plaques and the expression of cognitive deficits (Shemer et al., 2006; Buskila et al., 2013; Goutagny et al., 2013; Latif-Hernandez et al., 2020). This aim toward the imperious need to focus the studies and subsequent interventions on earlier-as-possible time points during the disease progression. However, despite the large amount of research in the brain plasticity field and the huge efforts of the scientific community, the mechanisms involved in the driving events underlying the functional loss-of-plastic processes during AD progression are unknown. More comprehensive studies could help to identify strategies directed to prevent the amplification of the Aβ42 toxicity mentioned above. In addition, such missing mechanistic data leads to current controversies with regards to the expression or loss of some forms of plasticity during AD progression compared to the early disturbances on synaptic transmission. While there is increasing evidence of functional disruption of neuronal networks activity early on AD progression (prior to amyloid depositions; Goutagny et al., 2013; Mondragón-Rodríguez et al., 2018), it is intriguing that some forms of plasticity appear to be affected only at later stages when the amyloid pathology and/or the cognitive impairment are established (Kimura et al., 2010; Crouzin et al., 2013; Latif-Hernandez et al., 2020; Garad et al., 2021). A better understanding of the processes underlying normal memory will drive to a better understanding of cognition-compromised disorders. Here we comment on the current knowledge of brain plasticity on AD and its relationship with brain oscillations and provide future perspectives from an integrative point of view (Figure 1).

## Synaptic Plasticity and Brain Rhythms in AD Models

In recent years, a wealth of data has been accumulated on the study of plasticity in AD by using a plethora of available and newly generated *in vitro, ex vivo*, and *in vivo* AD models as well as different electrophysiological approaches. This offers several advantages on the field toward the understanding of particular features of the pathology while brings a complex picture of the disease. Some of the diverse studies on plasticity are outlined in Table 1.

**TABLE 1:** Summary of studies on LTP in AD-related models showing the diversity of approaches and the studied time point during AD progression.

AD model	Plasticity protocol	Age of impairment studied/expression	Brain area/synapse	References
Cerebral microinjections of human naturally secreted Aβ42 oligomers to adult Wistar rats.	LT-HFS inducing protocol. *In vivo.*	NA	Hippocampal CA1 area.	Walsh et al., 2002
Acute Aβ42 application to wt mice brain slices.	Theta-nested gamma oscillations induced LTP. *Ex vivo.*	NA	Hippocampal CA1 area.	Park et al., 2020
Acute Aβ42 application to wt mice brain slices.	LTP: HFS protocol. *Ex vivo.*	NA	Hippocampal CA1 area.	Samidurai et al., 2018
Acute Aβ42 application to wt mice brain slices.	LTP: HFS protocol. *Ex vivo.*	NA	Hippocampal CA1 area.	Rammes et al., 2017
Acute Aβ42 application to wt mice brain slices.	LTP: TBS protocol. *Ex vivo.*	NA	Hippocampal CA1 area.	Zhang et al., 2017
Acute Aβ42 application to wt mice brain slices.	Optogenetic induced α7 nAChR-dependent tLTP	NA	Hippocampal CA1 area. (septal input stimulation)	Gu and Yakel, 2011
APPswe/PS1dE9 mice	STDP-LTP: pre-post inducing protocol. *Ex vivo.*	3.5 months old	Somatosensory cortex, layer 2/3.	Shemer et al., 2006
APP (KM670/671NL) / PS1 (L166P) mice	STDP-LTP: pre-post inducing protocol. *Ex vivo.*	6 months old	Hippocampal CA1 area.	Garad et al., 2021
AppNL-G-F mice	LTP: HFS protocol. *Ex vivo.*	3–4 months old	Medial prefrontal cortex	Latif-Hernandez et al., 2020
AppNL-G-F mice	Late-LTP: TBS protocol. *Ex vivo.*	6–8 months old	Hippocampal CA1	Latif-Hernandez et al., 2020
5xFAD mice	LTP: TBS protocol *Ex vivo.*	6 months old	Hippocampal CA1	Crouzin et al., 2013
5xFAD mice	LTP: TBS protocol *Ex vivo.*	6 months old	Somatosensory cortex layer 5.	Crouzin et al., 2013
J20 mice	LTP: TBS protocol *Ex vivo*	4–6-month-old	Medial perforant path to Dentate Gyrus granule cell synapses	Zhang et al., 2017
3xTg mice	LTP: HFS protocol *Ex vivo*	Postnatal day 7	Hippocampal CA1	Joseph et al., 2019
3xTg mice	LTP: HFS protocol *Ex vivo*	14–16-month-old	Hippocampal CA1	Joseph et al., 2019

**NA, not applicable.**

### Studies on Acute Aβ42 Application to Mouse Brain Slices and Cerebral Microinjections

In preclinical AD models, Aβ deposition is primarily caused by the increased production of Aβ > 40. As such, Aβ42 is the Aβ specie that results in plaque deposition. Aβ40 is comparatively benign and may even be protective (reviewed in Karran et al., 2011; Sasaguri et al., 2017). It is established that acute effects of either preincubation with- or wash-in Aβ42 (*ex vivo* AD-model) or hippocampal Aβ42 injections *in vivo* likely serve as a feasible prototype for cognition-relevant neuronal network dynamics in AD, since several synaptic and neuronal AD features has been replicated (Pena-Ortega et al., 2012; Kurudenkandy et al., 2014; Balleza-Tapia et al., 2018; Andrade-Talavera et al., 2020, 2021; Park et al., 2020).

However, it has been observed that acute Aβ42 application could induce a biphasic effect on neuronal networks consisting in an initial decrease of activity followed by overexcitation (Wang et al., 2009). Acute Aβ42 has also been shown to produce a transient decrease of network activity in cultured neuronal networks followed by a recovery (Görtz et al., 2009). These results likely depend on the conformation, time of exposure, peptide concentration used and possible compensatory mechanisms (see Peña-Ortega, 2013). A similar scenario is found for acute Aβ effects in studies on plasticity reporting that it dramatically disturbs LTP and LTD (Shankar et al., 2008; Li et al., 2011; Rammes et al., 2017; Samidurai et al., 2018), whereas other studies report that low physiologically relevant concentrations of Aβ promote LTP and memory (Palmeri et al., 2017). It has been observed that Aβ induces dysfunction of glutamatergic neurons impairing septum rhythmicity which may negatively affect hippocampal rhythmogenesis (Leão et al., 2012). In fact, septal cholinergic input is crucial for setting hippocampal theta rhythm (Buzsáki, 2002) and is severely impaired in AD (Terry and Buccafusco, 2003; Mondragón-Rodríguez et al., 2019). Accordingly, acute Aβ impairs carbachol-induced theta-gamma interaction in the hippocampal CA3 area (Andrade-Talavera et al., 2021) and prevents α7 nAChR-dependent LTP and short-term depression in CA1 (Gu and Yakel, 2011). By contrast, in the latest study, mAChR-mediated LTP showed to be relatively resistant to Aβ (Gu and Yakel, 2011). Another approach used to determine the neurotoxic effects of Aβ in LTP induction has been the cerebral injections of naturally secreted human Aβ oligomers. Cerebral microinjection of cell medium containing these oligomers and abundant Aβ monomers but no amyloid fibrils markedly inhibited HFS-induced LTP of hippocampal CA1 area in rats *in vivo* (Walsh et al., 2002).

### AD Animal Models

3xTg AD mice model exhibits hypo-excitable synaptic transmission, reduced paired-pulse facilitation (PPF), and normal LTP at 7 days old in the hippocampal CA1 area in response to HFS of Schaffer collaterals. By contrast, at 14–16 months old, the same model exhibits hyper-excitable synaptic transmission, enhanced PPF, and unstable LTP (Joseph et al., 2019). Interestingly, synaptic plasticity is impaired at the medial perforant pathway to DG granule cell synapses but not at the Schaffer collateral to CA1 pyramidal cells of 4–6 months old J20 AD mice model (Zhang et al., 2017). In 6 months old 5xFAD mice (another transgenic model of AD), LTP in the layer 5 of the somatosensory cortex is more severely impaired than LTP triggered in the CA1 area of the hippocampus (Crouzin et al., 2013). 5xFAD AD mice exhibits massive Aβ deposition in both regions but with different features: in cortical areas a majority of Aβ deposits comprise a dense core surrounded by a diffuse corona while such kind of Aβ deposition is less frequently observed in the hippocampus (Crouzin et al., 2013). This study, conducted by applying a classical high-frequency stimulation LTP protocol, suggests that cortical plasticity is deficient in the 5xFAD model and that this deficit could be correlated with the proportion and morphology of Aβ plaques observed in these mice and area-specific alterations of the synaptic transmission and plasticity were observed. In the same model no disturbances in synaptic transmission have been observed at 4 months of age (Kimura et al., 2010).

5xFAD mice start to develop visible Aβ deposits as early as 2 months of age, consistent with their dramatically accelerated Aβ1-42 production. This Aβ deposition first emerges in the subiculum area of the hippocampus and in the layer 5 of somatosensory cortex, and then rapidly increases with age, spreading to fill much of the hippocampus and cortex by 9 months of age (Crouzin et al., 2013). In another commonly used AD mice model (APP/PS1), at 6 months old, it has been recently found in the CA1 region of the hippocampus that LTP magnitude is significantly reduced if the recorded CA1 pyramidal neuron is located in the vicinity of Aβ plaques (<200 μm), when using a STDP-induction protocol (t-LTP) (Garad et al., 2021). APP/PS1 AD mice model shows small, compact Aβ deposits that are found scattered in L2/3 already at the age of 3.5 months. Plaque appearance and distribution occur at later ages (Shemer et al., 2006). In pyramidal cells from the somatosensory cortex layer 2/3 of the same AD model, t-LTP is significantly decreased at 3.5 months old mice and abolished at 7 months old animals (Shemer et al., 2006).

By performing acute Aβ applications *ex vivo*, the authors proposed that soluble Aβ might trigger the decrease of synaptic plasticity in neocortical pyramidal cell networks during early stages of AD pathogenesis by preferentially targeting postsynaptic AMPA receptors (Shemer et al., 2006). In fact, plasticity impairment should be supported/driven by synaptic failure at some extent. In this regard, pyramidal neurons from cortical layer 5 of 5xFAD mice aged 8–12 weeks are structurally and morphologically normal for this age. However, synaptic deficits at this early time point have been reported preceding any structural dystrophy typical of older ages in this model (Buskila et al., 2013). Recently, an intact synaptic function and synaptic plasticity in the hippocampus of a novel AppNL-G-F mouse model has been observed at 3–4 months of age. By contrast, impairment of synaptic plasticity starts at 3–4 months and of basal synaptic transmission at 6–8 months in medial prefrontal cortex of AppNL-G-F mice (Latif-Hernandez et al., 2020).

### NMDAR in Plasticity and Network Performance

Many forms of plasticity, including some forms of STDP, require the activation of glutamate NMDA-type receptors (NMDAR) (Bender et al., 2006; Brasier and Feldman, 2008; Rodríguez-Moreno and Paulsen, 2008; Banerjee et al., 2009, 2014; Rodriguez-Moreno et al., 2010, 2011, 2013; Andrade-Talavera et al., 2016; Bouvier et al., 2018; Pérez-Rodríguez et al., 2019) and NMDAR antagonists predominantly increase network hypersynchrony *in vivo* (Hanson et al., 2020). Interestingly, enhancement of GluN2A-subunit-containing NMDAR, counteracts aberrant low-frequency oscillatory power that is tightly correlated with network hypersynchrony in a family AD mouse model (J20), reduces epileptiform discharges and improves cognitive functions (Hanson et al., 2020). In addition, LTP induced during the rising theta phase is NMDAR sensitive in the CA1 area of the hippocampus (Law and Leung, 2018). A permanent reduction of expression/activity of GluN2B-subunit-containing NMDAR has been found to counteract LTP impairment in the hippocampal CA1 area of the AD mouse model mAPP (Rammes et al., 2017). During the 5th postnatal week, a developmental switch occurs for STDP with the emergence of a presynaptic, NMDAR-independent form of t-LTP at hippocampal CA3-CA1 synapses (Falcón-Moya et al., 2020). In previous postnatal weeks, the same STDP protocol induces NMDAR-dependent t-LTD (Andrade-Talavera et al., 2016). However, the exact mechanisms underlying the switch in plasticity rules in other brain areas and synapses in healthy conditions and AD remain elusive. A possible relationship with oscillations has not yet been demonstrated.

### Glial Involvement in Promoting Network Activity or Its Disruption

In addition to neurons, glial cells are also involved in the control of synaptic transmission, synaptic plasticity and neuronal synchronized activity (Min and Nevian, 2012; Rodriguez-Moreno et al., 2013; Andrade-Talavera et al., 2016; Perea et al., 2016; Szepesi et al., 2018; Frere and Slutsky, 2018; Mederos et al., 2018; Navarrete et al., 2019; Pérez-Otaño and Rodríguez-Moreno, 2019; Pérez-Rodríguez et al., 2019; Falcón-Moya et al., 2020). Recent studies suggest that chronic changes in neuronal activity bi-directionally regulate microglia function and amyloid depositions in AD mouse models (reviewed in Szepesi et al., 2018). Microglia and neurons make transient physical contacts regulated by neuronal activity and sensory experience in different vertebrate species (Bachiller et al., 2018; Szepesi et al., 2018). Moreover, microglia plays a central role commanding neuroinflammation in AD. Sodium butyrate (NaB), which reduces the secretion of pro-inflammatory cytokines, has recently been shown beneficial rescuing effects over impaired LTP and cognition in 2 months old-treated 5xFAD mice (Jiang et al., 2021). In an interesting study, *in vivo* stimulation of fast-spiking interneurons at γ frequency altered microglia morphology inducing a shift from pro-inflammatory to phagocytic phenotype that resulted in a significant reduction of the amyloid load in 5xFAD mouse model and improved network performance (Iaccarino et al., 2016). Also, optogenetic stimulation of parvalbumin interneurons at 40 Hz restores hippocampal slow gamma oscillations amplitude, and phase-amplitude coupling of the J20 AD mouse model, resulting in the rescue of spatial memory in mice despite significant plaque deposition (Etter et al., 2019). Overall, it has been observed that pathological triggers and drivers such as aberrant peptides accumulation, microglia-mediated inflammation and astrocytes dysfunction underlie spike-timing precision deterioration and neuronal network collapse which lead to cognitive impairment typical of AD (see Frere and Slutsky, 2018). However, the mechanisms governing timing-dependent plasticity windows relative to brain rhythms during normal brain development and disease progression are yet to be deeply elucidated.

## Discussion

The exact cellular mechanisms at the root of plasticity changes during brain development in the course of the progression of aberrant network activity leading to cognitive dysfunction in AD remain elusive. Such knowledge is crucial to identify suitable targets for therapeutic attempts at prevention of or rescue from the detrimental effects of cognition-compromising triggers and drivers. Despite the fact that familial AD (FAD) contribution to the overall burden of AD cases could be considered negligible, its discovery has boosted the generation of a diverse transgenic mouse models carrying a combination of human mutations that triggers pathogenic events sharing commonalities with human sporadic AD, such as Aβ deposition and progressive cognitive decline.

Accordingly, some features of the mentioned models give rise to differences on timely physiological deviations between models, particularly if the focus is turned onto earlier time points of the disease progression. The contribution of Walsh et al. (2002) performing microinjections of human secreted oligomers and abundant Aβ monomers but no amyloid fibril offered an advantageous paradigm compared with the available models up to the date of the study. It brought the ability to study the effects of biochemically defined assembly forms of naturally produced human Aβ at physiological levels, in the absence of any confounding effects of amyloid precursor protein (APP) overexpression.

Despite either the controversy or the ample commonalities found among the current studies in diverse AD models, such studies suggest but do not dissect out the earliest changes in the neuronal networks dynamics at the root of the progressive deterioration of timing precision activity. Although APP-overexpressing transgenic mice have been important tools in AD research, concerns exist regarding the interference of the non-physiologically high levels of APP and its proteolytic fragments with normal brain function. Such APP processing products are not increased in human AD (Sasaguri et al., 2017), and the creation of artificial phenotypes represents another concern that could underly the current controversies. The use of a novel knock-in mouse model could overcome this problem as it utilizes the endogenous mouse APP gene carrying the Swedish KM670/671NL (NL), the Artic E693G (G) and the Beyreuther/Iberian I716F (F) mutations with a humanized Aβ sequence (AppNL-G-F) (Saito et al., 2014). This novel mouse model starts to show amyloid plaque formation at 3 months of age and behavioral impairment and neuroinflammation at 6 months of age. However, a recent study from the researchers who generated the model observed that plasticity impairment does not start much earlier compared with the first generation of FAD mouse models (Latif-Hernandez et al., 2020).

A widely number of different protocols are used to induce LTP such as theta burst stimulation (TBS), which is based on the hippocampal rhythm within theta band frequency (4–8 Hz) (Larson and Lynch, 1989). Compared to HFS protocol (100 Hz, 1s stimulation) TBS may be closer to some physiological conditions (Latif-Hernandez et al., 2020). However, it does not fully overcome the existing need of protocols considering the coincident activity (pre- and postsynaptically) with the underlying brain state such as ongoing neuronal networks rhythms. Recently, the work of Park et al. (2020) reveals a plausible approximation to the *in vivo* phenomena showing that theta-nested gamma oscillations induced LTP is impaired in the hippocampal CA1 area of slices treated with acute Aβ42. In the study, optogenetic activation of SST has successfully restored theta-nested gamma oscillations-induced LTP from Aβ42-induced impairment. Finally, the emergence and termination of some forms of plasticity during critical periods has been generally addressed in quiescent states out of considering a more physiological scenario where external inputs such as ongoing neuronal networks oscillations command the sign of plasticity as it may occurs *in vivo*. Furthermore, despite the clear contribution of Aβ42 to the pathology, putative source of other mediators of the functional collapse remains elusive or mainly consider involvement of astrocytes while microglia involvement remains poorly studied.

Notably, the notions on experimental plasticity (animal models, *in vitro* and *ex vivo*) may find a plausible target for plasticity studies in humans within the transcranial magnetic stimulation (TMS) technique. TMS could cross-validate diverse features of such experimental advances regardless possible differences between the mechanisms underlying TMS-induced LTP-like or LTD-like and mechanisms supporting experimental plasticity (see Rawji et al., 2020). Electroencephalography (EEG) has shown to be valuable as predictive translational biomarker for AD (Stoiljkovic et al., 2018). The combination of TMS with EEG bring a non-invasive method for direct and timely exploration of excitability and connectivity properties of the stimulated cortical area which could reveal functional connectivity in healthy and pathological conditions (see Nardone et al., 2021). Additionally, it could represent a powerful diagnostic and following-up tool (Koch et al., 2011) with therapeutic potential (see Mondragón-Rodríguez et al., 2016). TMS has revealed LTP impairment in AD patients (Di Lorenzo et al., 2016, 2018, 2019) showing predictive potential for conversion to dementia in patients with impaired LTP (Di Lorenzo et al., 2020). Another technique that has promisingly emerged in the past 10 years is the Deep Brain Stimulation (DBS). Despite it is an invasive neurosurgical technique it has shown a certain positive effect in animal AD models as well as in AD patients. However, controversial results exists probably due to refinement of the approach in AD (see Luo et al., 2021). There is a need of standardize and coordinate acquisition and analysis protocols in a user-friendly way within larger cohort populations in order to incorporate electrophysiology as a part of the clinical criteria of AD (Yener and Başar, 2013).

Back to basic research in animal models, it is tempting to hypothesize that some forms of synaptic plasticity and their developmental expression could be different from the ones studied with current protocols without including the external inputs such as rhythmic network activity and that such kind of functional plastic changes appears disrupted prior to the expression of cognitive impairment on AD progression. Moreover, while synaptic plasticity induced with high frequency stimulation protocols is widely accepted and useful, STDP protocols encompass more physiological features of the neuronal dynamics underlying brain plasticity phenomena. Going beyond, the need for tools that contribute to early diagnosis of AD reveals the needs to explore the mechanisms underlying the normal network development and the progressive deterioration of cognition-relevant neuronal dynamics. Future work should then increase the efforts toward the convergent study of plasticity with protocols closer to physiological conditions to dissect out the modulating contribution of brain states (network rhythms/diverse synaptic inputs) to the emergence, loss, sign and forms of synaptic plasticity across brain development in healthy condition, thus helping to set the basis for the comprehensive study of plastic processes during the progression of cognitive-compromised disorders, majorly AD. Finally, regardless controversies, from this complex picture a promising horizon is rising targeting and based on basic, clinical and therapeutic AD research. Probably the commonalities among the diverse methods and models have set the basis for the current approaches. The success in restoring neuronal dynamics by optogenetic stimulation of key neuronal populations in AD models and reliability of TMS in human neurological disorders offers a suitable scenario where putative therapeutic tools could emerge.

## Data Availability Statement

The original contributions presented in the study are included in the article/supplementary material, further inquiries can be directed to the corresponding author/s.

## Author Contributions

Both authors listed have made a substantial, direct and intellectual contribution to the work, and approved it for publication.

## Conflict of Interest

The authors declare that the research was conducted in the absence of any commercial or financial relationships that could be construed as a potential conflict of interest.
